# Large language models as cognitive shortcuts: a systems-theoretic reframing beyond bullshit

**DOI:** 10.3389/frai.2026.1681525

**Published:** 2026-02-13

**Authors:** Murat Sariyar

**Affiliations:** School of Engineering and Computer Science, Bern University of Applied Sciences, Bern, Switzerland

**Keywords:** bullshit, ChatGPT, Deleuze, LLM, Luhmann, simulacra

## Abstract

**Introduction:**

Large Language Models (LLMs) are often framed through metaphors such as “bullshit” or “stochastic parrots,” emphasizing missing grounding, belief, or intention. While rhetorically powerful, these framings obscure how LLMs are used for sense-making, ideation, and communication. We reframe LLMs as Operators for General Cognitive Shortcuts (GECOS) within techno-semiotic assemblages.

**Methods:**

We develop a functional model by integrating concepts from Luhmannian systems theory, Deleuzian ontology, and minimally from Husserlian phenomenology. Using conceptual analysis as functional–comparative synthesis, we analyze human–LLM interaction without attributing agency, belief, or understanding to the model.

**Results:**

GECOS explains LLM usefulness as communicative complexity reduction: models generate connectable continuations by approximating second-order expectations (“what is expected to be expected”), enabling interactional continuity without reference to truth or intention. Via Luhmann’s contingency formula, LLMs help users navigate uncertainty through procedurally plausible coherence.

**Discussion:**

The framework shifts attention from ontological debates about “understanding” to the operational role of LLMs in distributed sense-making. It also highlights risks: overreliance, emotional projection, and normative flattening when connectability substitutes for justification.

**Conclusion:**

GECOS offers a non-anthropomorphic alternative to deficit metaphors by modeling LLMs as pragmatic operators that sustain communicative momentum and enable workable continuations in complex socio-technical environments.

## 1 Introduction

Large language models (LLMs) are increasingly discussed through metaphors intended to make their behavior immediately intelligible. Terms such as *hallucination*, *confabulation*, *bullshit*, or *stochastic parrots* have become common descriptors for false or misleading outputs (Bender et al., 2021; Matthews, 2025; Smith et al., 2023; Tigard, 2025). While rhetorically effective, these metaphors often import cognitive, phenomenological, or moral assumptions that do not cleanly apply to systems that neither perceive, believe, nor intend. To describe an LLM’s false output as a hallucination, for instance, presupposes a breakdown between perception and reality—a failure of sensory–intentional coordination (Shanahan, 2024b). Yet LLMs have no sensory access, no phenomenal field, and no world-relative belief states that could be misaligned in this way (Bender and Koller, 2020; Harnad, 2025). Similarly, framing LLM outputs as confabulation or bullshit presumes agency, epistemic stance, and motivational indifference to truth (Frankfurt, 1986; Tigard, 2025), whereas contemporary models generate sequences of signs through self-supervised learning over large text corpora (Zhou et al., 2021) and subsequent alignment procedures such as reinforcement learning from human feedback (Ziegler et al., 2020).

The persistence of these metaphors nonetheless signals a genuine problem. LLMs do not encode meaning in the formal-semantic sense (Montague, 1970), but they reliably produce outputs that users experience as coherent, responsive, and usable. Technically, this coherence arises from high-dimensional vector spaces shaped by latent relational patterns (Piantadosi and Hill, 2022; Sahlgren and Carlsson, 2021) and from probabilistic convergence on contextually appropriate continuations (Manning et al., 2020). Empirical work suggests increasingly complex internal representations, including nonlinear dynamics and latent structural features (Piantadosi, 2021; Søgaard, 2023). Yet purely technical descriptions do not explain why LLMs have become pervasive in practices of drafting, ideation, explanation, and coordination—nor why users routinely rely on them in situations where correctness, intention, or grounding are explicitly bracketed. The conceptual gap lies not in architecture but in interaction: how can systems without understanding nonetheless function as stable components of sense-making processes?

Recent philosophical discussions sharpen this tension. Shanahan emphasizes that bare LLMs are fundamentally model-free in a strong epistemic sense: they cannot measure their outputs against external reality and revise beliefs accordingly, a capacity central to robust notions of belief and understanding (Shanahan, 2024a). Tigard similarly argues that what distinguishes LLM outputs is not merely falsity but fakery: they perform epistemic posture without commitment (Tigard, 2025). These critiques are compelling insofar as they resist anthropomorphism. However, they do not yet account for how LLMs nevertheless stabilize communicative interaction in practice. At the same time, the socio-technical context of LLM deployment is changing rapidly. LLMs are increasingly embedded in infrastructures that include retrieval systems, verification pipelines, APIs, plugins, and live data sources, which constrain output space and orient generation toward procedural verifiability (Davis and Aaronson, 2025). In such environments, truthfulness is no longer primarily a matter of internal belief or intention but of architectural coupling and evaluation regimes (Fisher, 2024). This shift renders purely deficit-oriented metaphors increasingly inadequate, even if they remain influential in public and scholarly discourse (Hicks et al., 2024).

What is missing is a conceptual framework that neither treats LLMs as quasi-subjective agents nor dismisses them as epistemically hollow simulators but instead captures how they operate within communicative systems. This paper proposes such a framework by introducing GECOS (General Cognitive Shortcuts). GECOS is not a theory of what LLMs are, nor a claim about their intelligence or understanding. Rather, it is a functional lens that describes what LLMs do in interaction: they reduce communicative complexity by providing shortcuts that approximate what a competent participant would plausibly contribute next in a given situation. In doing so, they functionally approximate second-order expectations (i.e., they generate continuations that fit what is typically expected to be expected in a given communicative situation) without beliefs, intentions, or world-grounded semantics, yielding connectable coherence rather than epistemic commitment. This does not preclude truth-apt outputs, especially under retrieval or verification constraints; however, the default success condition in many uses is pragmatic connectability.

This reframing shifts the focus from ontological debates about machine understanding to the dynamics of distributed sense-making. Users do not primarily interact with LLMs as epistemic authorities or intentional interlocutors, but as operators that offload the labor of phrasing, elaboration, and stylistic alignment. In many contexts—drafting emails, outlining arguments, brainstorming ideas, rephrasing explanations—the value of an LLM output lies not in its truth but in its usability: its capacity to sustain interaction, enable continuation, and maintain coherence under uncertainty. GECOS names this function explicitly and thereby offers an alternative to metaphors that frame LLMs mainly in terms of epistemic failure.

The approach also differentiates itself from recent Luhmann-inspired accounts that frame LLMs as quasi-autopoietic or already socially coupled systems (Lovasz, 2023; Zönnchen et al., 2025). While these accounts clarify important features of communication theory, they tend to treat human–LLM interaction as more closed than it actually is, overlooking its provisional and easily disrupted character. The present account instead emphasizes a pre-systemic layer of expectation alignment: interaction becomes possible when outputs are connectable to user expectations and can be iteratively refined, even if the system itself does not participate in self-referential meaning production. In this respect, the paper aligns with critiques of anthropocentric framings such as Dennett’s intentional stance (Dennett, 1983), while insisting that a functional account of interaction is still required.

By introducing GECOS, this paper contributes a meta-structural lens for understanding LLMs as operators within techno-linguistic environments. Rather than asking whether LLMs understand, believe, or know, it asks how they enable continuity, coherence, and creative momentum in communicative practice, and at what cost. This shift foregrounds both the productive role of LLMs in navigating complexity and the risks that accompany reliance on procedural plausibility, including overreliance, emotional projection, and normative flattening (Liang et al., 2024). The sections that follow elaborate this framework, situating it philosophically and illustrating how GECOS reshapes how we evaluate, design, and work with large language models.

## 2 Conceptual method and theoretical orientation

This paper employs conceptual analysis, understood not as definitional clarification or conceptual genealogy, but as a functional–comparative synthesis aimed at explaining how interaction with LLMs stabilizes sense in practice despite the absence of belief, intention, or semantic grounding. The guiding methodological question is not what LLMs are, but how interaction with LLMs functions within communicative systems. Accordingly, the analysis proceeds by identifying a target phenomenon, specifying contrast classes, selecting minimal theoretical resources, and evaluating conceptual proposals against explicit adequacy constraints.

The target phenomenon is the empirical stability of human–LLM interaction: users routinely experience LLM outputs as coherent, responsive, and usable, even while recognizing that the system lacks understanding, world-access, or epistemic commitment (Bender and Koller, 2020; Shanahan, 2024a). Existing explanations either remain at the level of technical description (e.g., distributional semantics, token prediction) or rely on metaphorical framings that presuppose cognitive capacities the system does not possess (hallucination, confabulation, bullshit) (Bender et al., 2021; Tigard, 2025). The methodological task is therefore to develop a conceptual model that accounts for this interactional stability without anthropomorphic projection.

As a contrast class, two dominant families of approaches are used. The first includes deficit-based metaphors that frame LLM outputs primarily in terms of epistemic failure or deception (Bender et al., 2021; Hicks et al., 2024). While diagnostically useful, these metaphors obscure how LLMs are nonetheless productively integrated into everyday communicative practice. The second includes approaches that attribute quasi-agential or quasi-systemic status to LLMs, for example by interpreting them as socially coupled or proto-autopoietic systems (Lovasz, 2023; Zönnchen et al., 2025). These accounts risk overstating systemic closure and underestimating the fragility and asymmetry of human–LLM interaction. GECOS is developed explicitly in contrast to both families.

Theoretical resources for the following are drawn selectively and minimally from three traditions, each contributing a specific functional insight rather than a comprehensive ontology. From Luhmannian systems theory, it takes the idea that communication does not transmit inner meanings but stabilizes expectations through recursive selection under conditions of contingency (Luhmann, 1981, 1996). Meaning, in this view, arises from the difference between what is actualized and what could be otherwise, and contingency formulas serve to render this openness manageable. From Husserlian phenomenology, the paper adopts a minimal insight about experience: dialogic fluency and timely responsiveness can elicit the sense of being addressed even in the absence of a genuine subject, because intentional expectations are triggered by recognizable response-forms rather than by access to inner states (Bernet et al., 2005; Husserl, 1977). From Deleuzian ontology, it draws a conception of sense as an incorporeal event or surface-effect—real in its capacity to reorganize trajectories of thought and action, yet independent of reference or intention (Deleuze, 1990, 1995). Together, these perspectives allow us to describe LLM outputs as structurally sense-producing without attributing subjectivity to the model.

These resources are synthesized by a functional equivalence principle: concepts are retained only insofar as they explain the same interactional outcome (how human–LLM exchanges achieve connectable coherence that can be taken up, revised, and continued) without introducing anthropomorphic assumptions about model agency, belief, or understanding. Using this principle matters because it lets us translate across Luhmann and Deleuze at the level of what their concepts do (the role they play in explaining interaction), rather than forcing them into a single shared ontology. On this basis, we propose GECOS as a parsimonious functional description that satisfies these constraints: LLM outputs act as shortcuts that reduce communicative friction by approximating second-order expectations about what a competent participant would plausibly contribute next, thereby stabilizing sense by keeping communication connectable and ongoing.

The resulting framework is evaluated against three explicit adequacy constraints. First, non-anthropomorphic adequacy: the model must not rely on attributions of belief, intention, or understanding to the LLM (Dennett, 1983; Shanahan, 2024b). Second, explanatory gain: the framework must explain why LLM outputs are experienced as usable and stabilizing in interaction, something deficit metaphors fail to do. Third, action-guiding yield: the framework should generate implications for evaluation, design, and responsible use, for instance by clarifying when reliance on procedural coherence becomes epistemically risky (Davis and Aaronson, 2025; Fisher, 2024). This methodological approach does not aim at empirical generalization. Rather, it provides a conceptual lens that renders observable interactional patterns intelligible and generates hypotheses that can be tested in future empirical work. The following sections apply this lens to the phenomenological structure of interaction, the systemic dynamics of expectation, and the ontological status of LLM outputs, culminating in the GECOS framework.

## 3 Social system theory and LLMs

Niklas Luhmann’s theory of social systems offers a particularly suitable framework for analyzing interaction with LLMs because it conceptualizes communication without presupposing shared understanding, consciousness, or intentional alignment. In contrast to subject-centered theories of meaning, Luhmann treats communication as an autonomous process that stabilizes itself through recursive selections. What matters is not whether participants “understand” one another internally, but whether communication continues in a way that remains connectable for subsequent contributions. This shift is crucial for making sense of LLM interaction, where continuity and coherence are achieved despite the absence of subjectivity on the model’s side. The attraction of Luhmann here is not that LLMs are social systems in his strong sense, but that his framework provides vocabulary for describing how order can emerge from contingency through expectation management rather than inner comprehension.

At the core of Luhmann’s theory lies the concept of double contingency: communication begins under conditions in which each participant knows that the other could respond otherwise and must therefore orient their own behavior toward this openness (Luhmann, 1981, 1996). In human interaction, double contingency is resolved neither by prediction nor by shared inner states, but by the formation of expectations about how expectations will be handled, i.e., by developing reliable patterns for uptake, repair, and continuation. Communication stabilizes when participants act as if their contributions will be taken up in a recognizable way, even if they cannot know this in advance. Importantly, this mechanism does not require transparency of motives or beliefs; it only requires that utterances remain connectable and that deviations can be repaired without collapsing the interaction. In Luhmann’s terms, this is the minimal condition under which communication can recursively reproduce itself as communication.

LLM interaction does not instantiate double contingency in its full sense. LLMs do not possess behavioral freedom, nor do they entertain expectations of their own. However, this does not render the concept irrelevant. On the contrary, it highlights a functional asymmetry: LLMs generate outputs that simulate responsiveness under contingency, while users adapt their behavior as if such contingency were operative. The interaction stabilizes because users treat the system’s outputs as plausible continuations within an assumed communicative situation, and because the system’s outputs are statistically tuned to be taken up as such continuations. What is reproduced here is not mutual openness, but the form of expectation alignment that typically resolves contingency in social interaction. In other words, the LLM supplies a highly elastic “next move” generator, and the user supplies the normative and pragmatic orientation that makes those moves meaningful within a situation.

This becomes clearer through Luhmann’s distinction between behavioral expectations and expectation-expectations (Luhmann, 2013b). Behavioral expectations concern anticipated actions (“I expect you to reply politely”), whereas expectation-expectations are second-order and reflexive (“I expect that you expect me to reply politely”). Only the latter enable interaction under genuine contingency, because they allow participants to orient themselves toward the anticipated orientation of the other rather than toward prefixed rules. In human–LLM interaction, users quickly shift from the first to the second mode. When an LLM produces an unsatisfactory output, users rarely interpret this as a violation in a normative sense; they do not “blame” the system as they might blame a human interlocutor. Instead, they rephrase prompts, add constraints, and supply missing context, treating the failure as a misalignment of the interactional frame rather than as a breach of commitment. This behavior indicates that users treat the interaction as a recursive calibration problem: they attempt to elicit outputs that would be connectable within the expectations they attribute to a competent participant in that situation (Luhmann, 1998).

Luhmann’s concept of the contingency formula further clarifies this dynamic (Luhmann, 2013a). In modern societies, communication is confronted with the awareness that everything could be otherwise, and that no ultimate foundation (metaphysical, moral, or epistemic) secures meaning once and for all. Contingency formulas do not eliminate this openness; they render it manageable by framing a particular selection as provisionally acceptable, thereby enabling continuation without requiring final justification. Classical examples include references to divine will, legal authority, or scientific method. What matters is not their truth but their capacity to stabilize expectations and coordinate how alternatives are handled. GECOS can be understood as such a technologically mediated contingency formula: it does not resolve uncertainty by reference to truth or authority, but by providing procedurally plausible continuations that keep communication moving and reduce friction when users face an overload of possible next moves.

Seen in this light, LLM outputs function less as answers than as connective proposals. They do not close down alternatives but present selections that are easy to accept, revise, or discard, often in a style that anticipates the norms of the communicative setting. This explains why users often experience LLMs as helpful even when outputs are known to be approximate, generic, or occasionally incorrect: the system supplies a scaffold for continuation that the user can inhabit, modify, and own. The function is not epistemic closure but interactional momentum, an enabling of further selections. By lowering the threshold for acceptable continuation, LLMs reduce communicative friction when the goal is not primarily truth but continuation, coordination, or usable form—e.g., producing a tactful refusal email, turning messy meeting notes into an agenda with action items, generating three alternative framings for a grant pitch, drafting a lesson-plan outline, or proposing first-pass titles and structure for a report—so that uncertainty about “what to say next” does not stall action.

This system-theoretic perspective also clarifies why anthropomorphic interpretations are both tempting and misleading. Because LLM outputs successfully align with expectation-expectations, they mimic the functional role typically played by conscious interlocutors in communication: they “respond” in a way that invites further response. Yet attributing beliefs or intentions to the system adds no explanatory value and risks reintroducing the very assumptions the analysis aims to avoid. What matters is the structural coupling between user expectations, prompt refinement, and output plausibility, an interactional loop in which the user supplies goals, norms, and responsibility while the model supplies connectable continuations. Luhmann’s framework is useful precisely because it allows us to describe this coupling without smuggling in an inner subject, as can happen in approaches that rely on intentional stance interpretations.

Finally, this analysis foregrounds a normative implication that is easy to miss in purely technical or purely metaphorical debates. Because GECOS operates by stabilizing communication through procedural plausibility rather than epistemic commitment, it carries an inherent risk of normative flattening: connectability can become a surrogate for justification. If communicative success is increasingly measured by how smoothly an output enables continuation, distinctions between well-founded, weakly grounded, and merely fluent contributions may erode, especially in settings where users are incentivized to prioritize speed over scrutiny. Luhmann’s theory does not resolve this risk, but it renders it intelligible: systems that rely heavily on contingency formulas trade epistemic depth for stability and manageability. Understanding LLMs as operators of such formulas makes it possible to analyze both their productivity and their hazards without moralizing metaphors or inflated ontological claims.

In sum, Luhmann’s theory provides conceptual tools to explain how interaction with LLMs can stabilize sense without shared understanding. By simulating second-order expectations and functioning as a contingency formula, LLMs enable communicative continuation under conditions of radical uncertainty, while shifting responsibility for norms and truth assessment onto the user and surrounding infrastructures. This system-theoretic grounding prepares the way for the next step of the argument: an ontological clarification of how such stabilization can occur without reference, intention, or depth, which we address through Deleuze’s concept of sense, event, and simulacrum.

## 4 Sense, simulacrum, and quasi-causality in LLM interaction

The Luhmannian account clarifies how communication can stabilize without shared understanding, but it does not yet explain why LLM outputs can feel senseful and trajectory-changing even when they are not anchored in belief or reference. To address this layer, we turn to Deleuze’s ontology of sense and the simulacrum. The aim is not to apply Deleuze as a grand metaphysical framework, but to extract a lean conceptual vocabulary for describing a phenomenon that is otherwise hard to name: LLM outputs often function as real events in discourse—shifting tone, reframing problems, unblocking thought—without operating as truth-apt representations or intentional expressions. Deleuze provides a way to describe this efficacy without smuggling in subjectivity.

For Deleuze, sense is not primarily a property of propositions understood as representations, nor a psychological state inside a speaker. Sense is an incorporeal event: a surface-effect that arises where expressions, contexts, and differential relations meet, organizing what can be said, felt, or done without being reducible to bodily causes or inner intentions (Deleuze, 1990). This is precisely the register in which many LLM interactions occur. When an LLM helps a user reframe a resignation message, restructure an argument, or find a more tactful formulation, the crucial effect is often not informational accuracy but a shift in the situation’s “sayability” and practical orientation. The output reorganizes a discursive field—what counts as the next plausible move, what tone becomes available, which implication becomes salient. Even when the model has no world-relative belief, the interaction can generate an event of sense: a new surface configuration that enables continuation.

This Deleuzian view also helps avoid a common confusion. One might think that if LLMs lack reference and intention, their outputs must be ontologically “hollow.” Deleuze’s concept of sense makes room for a different claim: an output can be real in its effects while remaining independent of inner depth. Sense is real as an event precisely because it operates by reorganizing relations at the surface of language—through contrast, variation, and the activation of latent patterns—rather than by mirroring an external world (Badiou, 2007). In LLM interaction, the model’s statistical capacities become relevant not as a substitute for understanding but as a generator of structured variation. The output is often useful because it introduces a small displacement (of wording, framing, order, register) that changes how a situation is navigated. In that way, the model functions as an operator of sense events, even when it cannot “mean” in the traditional sense.

The notion of *quasi-causality* sharpens this point (Roffe, 2017). Quasi-causality designates a mode of effectuation that is neither efficient physical causality nor intentional agency, but causal-looking efficacy that arises from relational configurations at the surface. A classic illustration is the wound: the knife causes a physical cut, but the event of being wounded is an incorporeal transformation that reorganizes narrative, identity, and temporal orientation. One is not merely injured; one becomes “wounded” in a sense that changes what can be said, expected, or done. LLM outputs often operate quasi-causally in this manner. They do not cause effects by representing knowledge or by intending outcomes; they produce effects by reconfiguring discursive relations, by offering a formulation that a user can take up, that changes the conversational trajectory, or that shifts a decision threshold. In this register, the efficacy of an LLM is not the efficacy of truth but the efficacy of form: the production of connectable discourse that allows a situation to move.

In this connection, Deleuze’s concept of the *simulacrum* can be situated. In classical accounts, a simulacrum is an inferior copy of an original (Baudrillard, 1994). Deleuze reverses this hierarchy: the simulacrum is not a deficient imitation but a productive appearance that operates without reference to an original and generates effects through difference (Deleuze, 1990). LLM outputs are plausibly described as simulacral in this Deleuzian sense. They often have the form of explanation, advice, or expertise, but they are not the expression of an epistemic subject and are not generated by world-involving commitments. They are produced through differential relations within linguistic corpora—patterns of usage, rhetorical templates, and stabilized discursive moves. The semblance is not simply a lie or a trick; it is a structural effect of learned distributions that can be taken up as meaningful within interaction. This is why deficit metaphors can mislead: what matters is less that the output may be false than that it can perform as if it were anchored, inviting uptake by virtue of its coherence.

At the same time, the socio-technical evolution of LLMs complicates a simple simulacrum diagnosis. Contemporary systems are often embedded in retrieval and verification architectures that constrain the output space through external checks (Davis and Aaronson, 2025; Fisher, 2024). This raises a question that many discussions flatten too quickly: does verification *abolish* simulation by reintroducing reference, or does it *reconfigure* simulation by adding procedural constraints? The latter interpretation seems more precise. Verification modules limit what the model can get away with, but they do not turn the model into a believer or a perceiver. They shift the conditions under which simulacral outputs are accepted. What emerges is not a transition from “mere text” to “grounded understanding,” but a coupling in which statistical generation is selectively tethered to external sources and filters (Harnad, 2003). The output may become more reliable in practice, yet the fundamental mode of production remains probabilistic continuation rather than world-involving commitment.

This is also why it is helpful to describe LLMs, especially in tool-using configurations, as operating within techno-semiotic assemblages—heterogeneous couplings of model, prompts, interfaces, retrieval systems, institutional norms, and user expectations (DeLanda, 2016). In such assemblages, sense is neither inside the model nor solely in the user; it is produced at the surface of interaction through iterative selection and uptake (Buchanan, 2017; Kleinherenbrink, 2020). The model contributes structured plausibility; the user contributes goals, constraints, and responsibility; the surrounding architecture contributes procedural checks and affordances. The result is a distributed production of sense events: outputs that can reorganize trajectories and stabilize continuations without requiring internal meaning on the model’s side (Hutchins, 1996). This is precisely the ontological niche that GECOS later names at the level of function.

Importantly, Deleuze’s framework also illuminates a limit condition. Not every coherent output generates an event. Sense, for Deleuze, requires differential tension and the possibility of transformation; when language collapses into redundancy or purely generic templates, discourse becomes ontologically inert, even if it remains grammatically correct. This maps neatly onto everyday LLM experience: users often report that outputs can become sterile when they merely restate the obvious, overfit common tropes, or foreclose alternatives. In such cases, the model still produces connectable text, but it fails to produce a meaningful event that changes the situation. This distinction is crucial for evaluation: a purely “smooth” continuation is not yet a sense event; the relevant measure is whether the output actually reorganizes a trajectory—whether it opens options, clarifies stakes, or enables action.

In sum, Deleuze provides the conceptual resources to describe how LLM outputs can be effective without being intentional or referential. Sense is an incorporeal surface event; quasi-causality names how such events effect real changes in orientation; and simulacrum captures the peculiar ontology of outputs that perform meaningfulness without original, speaker, or depth. When these outputs are coupled to verification infrastructures, simulation is not abolished but constrained and redirected. This ontological account complements the system-theoretic analysis of contingency by clarifying the mode in which LLM outputs operate: not as representations that mirror reality, but as surface configurations that can produce real events of sense. It thereby sets up the next step of the paper: the introduction of GECOS as a meta-structural lens that captures this functional role.

## 5 A new lens for LLMs: general cognitive shortcuts

The preceding analyses clarified two complementary points: first, that interaction can stabilize through expectation-management rather than shared understanding (Section 3), and second, that LLM outputs can function as events of sense—surface reconfigurations that change what becomes sayable and doable—without implying reference or intention (Section 4). GECOS (General Cognitive Shortcuts) integrates these insights into a single functional lens that stays at the level of operation. It does not claim that LLMs understand, nor does it reduce them to mere generators of defective representations. Instead, it specifies a recurrent interactional role: LLMs reduce communicative complexity by supplying connectable continuations that let users proceed when the next discursive move is uncertain, costly, or blocked. This orientation matters because it explains LLM uptake without importing cognitive capacities the model lacks.

GECOS captures a pattern that is easy to observe but hard to conceptualize with deficit metaphors: users primarily mobilize LLMs as form-generators under constraints. Many everyday tasks are not primarily informational but compositional—finding an acceptable phrasing, an intelligible structure, an appropriate register, a workable outline, a tactful tone. In such cases, the model’s value lies in retrieving and recombining socially stabilized discourse patterns so that users can select among viable options rather than invent the form from scratch. This is why distributional capacities become practically consequential without becoming “understanding”: the system can traverse discursive neighborhoods that humans recognize as fitting a situation, even when substantive correctness is not guaranteed (Manning et al., 2020; Piantadosi, 2021; Sahlgren and Carlsson, 2021). GECOS names precisely this offloading of rhetorical and organizational labor, and it thereby explains why LLM use often increases speed and fluency even when users remain epistemically cautious.

The “general” character of GECOS indicates that these shortcuts are portable across domains. They are not specialized heuristics grounded in expert competence, but broadly applicable capacities to generate plausible continuations across heterogeneous contexts. This generality is not merely a technical fact; it has a distinctive interactional consequence: it encourages users to treat the model as a universal relay for “the next move,” even where stakes differ dramatically. The same mechanism that supports brainstorming or drafting can also be drawn into settings where evidential standards are high. This is where the framework becomes diagnostic rather than celebratory: generality explains both the reach of LLMs and their tendency toward pattern-conformity and overgeneralization. It also helps interpret why tool-augmented systems matter: as LLMs are coupled to external sources, the shortcut does not become a belief-forming mechanism, but a procedurally constrained generator of continuations whose acceptability is increasingly shaped by architecture. This is the point at which “plausibility” becomes institutionally engineered.

GECOS also clarifies a division of labor that is often obscured by the conversational interface. The model supplies connectable continuations; the user supplies aims, constraints, and normative orientation. What looks like “collaboration” is better described as a sequence of selections in which the system offers a space of discursive candidates and the user authorizes one path through that space. This matters because it explains why responsibility tends to migrate toward the user across iterations: each refinement is a selection among alternatives, and selection is where agency resides. In that sense, GECOS reframes authorship as a gradient rather than a binary: the model can contribute phrasing and structure while the user contributes commitment, context, and uptake. This avoids both extremes—treating the model as a speaker or treating the user as a passive recipient—while remaining compatible with the asymmetry emphasized in systems-theoretic analyses of continuation and expectation.

Table 1 positions GECOS relative to two influential interpretive lenses that the paper aims to move beyond without dismissing. The bullshit framing correctly flags epistemic risk and the appearance of competence without commitment, but it is poorly suited to explain why LLM outputs are nonetheless integrated as productive components of communicative workflows. The simulacrum lens captures the ontological oddness of meaning-like effects without original reference, but it can remain at the level of status-description. GECOS complements both by shifting the question from “what kind of sign is this?” to “what kind of shortcut does this enable?” In doing so, it also reframes what counts as success: not truth in isolation, but the capacity to generate a continuation that can be responsibly taken up, edited, or discarded in context.

**Table 1:** Conceptual comparison of three metaphors used to describe LLMs: bullshit, simulacrum, and GECOS.

Dimension	Bullshit	Simulacrum	GECOS
*Core question*	Why is truth disregarded?	How can sense appear without an original?	How does output enable continuation?
*What it explains*	Epistemic risk/fakery	Ontological ambiguity of meaning-like effects	Everyday usability as a shortcut
*Relation to truth*	Indifferent stance	Displaced by surface effects	Procedurally constrained by context/tooling
*User role*	Recipient/target	Interpreter of appearances	Co-constructor and editor

Because GECOS focuses on operation, it is positioned to articulate risks in a way that connects directly to use. One risk is overreliance, where the ease of continuation encourages users to substitute procedural fluency for substantive evaluation, especially under time pressure or cognitive load. Another is normative flattening, where shortcut-driven composition pulls discourse toward dominant, high-frequency templates and away from idiosyncratic or dissenting expression. A third risk is a form of epistemic opacity: as outputs become smoother and as procedural checks are externalized into toolchains, it becomes easier to confuse workflow reliability with epistemic. These risks are not accidental side effects; they are structurally adjacent to the very feature that makes shortcuts attractive, namely, their capacity to reduce friction by offering readily connectable forms.

Finally, GECOS is meant to be action-guiding without pretending to be a complete theory. It suggests a shift in evaluation and design questions: from assessing isolated outputs toward assessing interactional dynamics: how systems shape what users accept as sufficient, how responsibility is distributed, and how procedural constraints modulate plausibility. This does not replace epistemic evaluation; it clarifies where epistemic evaluation tends to be displaced by workflow success. In that sense, GECOS is a diagnostic lens for the emerging ecology of language technologies: it helps specify when shortcuts are enabling (ideation, drafting, exploratory thinking) and when they become hazardous (high-stakes decision support without robust verification and user calibration). The next section makes this operational by illustrating how the shortcut function becomes visible in practice: how expectation alignment is produced, how continuations are stabilized, and where the framework predicts failure modes that invite empirical follow-up.

## 6 GECOS in practice: how cognitive shortcuts operate across contexts

To illustrate how GECOS functions in concrete interaction, this section examines several everyday use cases in which large language models are employed not as epistemic authorities but as operators for cognitive shortcuts. These examples are not presented as empirical case studies or evidence for causal claims. Rather, they serve a diagnostic purpose: they make visible how expectation alignment, responsibility shifting, and communicative stabilization occur in practice, and where the limits of shortcut-based interaction become apparent.

Consider first a *writing and coordination scenario*. A user asks an LLM to draft a brief message declining a collaboration request while maintaining a positive relationship. The initial output is typically fluent but generic. The user then asks for a warmer tone, a shorter version, or a version that emphasizes future openness. With each iteration, the model supplies alternative formulations that are immediately recognizable as viable within the social situation. What matters here is not that the model “knows” anything about the relationship or the stakes involved, but that it reliably generates phrasing that fits established norms of professional politeness. The shortcut lies in bypassing the need to search for acceptable wording oneself. At the same time, responsibility becomes increasingly explicit: the user selects, edits, and ultimately sends the message. The LLM enables continuation, but it does not own the act. GECOS captures this division of labor: the model supplies form; the user supplies commitment.

A second example comes from *conceptual or academic work*, such as outlining an argument or clarifying a dense passage of text. Users frequently prompt LLMs to “rephrase,” “summarize,” or “make this clearer”. In such cases, the model’s output is rarely final. Instead, it functions as a provisional restructuring of the material: highlighting implicit assumptions, making argumentative steps explicit, or proposing alternative framings. Even when the output is imperfect, it often succeeds in shifting the user’s perspective on their own text. The shortcut here is not merely linguistic but cognitive: by externalizing a possible reorganization, the model enables the user to see their own material differently. Again, this effect does not depend on the correctness of the rephrasing. What matters is that the output is *connectable*—that it offers a structure the user can accept, reject, or refine. In Deleuzian terms, the output operates as a sense event; in GECOS terms, it functions as a shortcut that reduces the effort required to explore alternative conceptual paths.

A third domain is *creative ideation*, such as brainstorming titles, metaphors, or narrative continuations. Here, the epistemic stakes are deliberately low, and the value of the interaction lies in variation rather than accuracy. Users often ask for “10 ideas” or “different angles,” fully aware that most outputs might be mediocre. Yet the shortcut remains effective because it expands the space of possibilities quickly and cheaply. The user’s task shifts from generation to selection. This is a paradigmatic case of GECOS at work: the model does not innovate in a strong sense, but it recombines existing patterns in ways that can trigger new associations for the user. The risk of overreliance is minimal here precisely because the shortcut is transparent and framed as exploratory. The example shows that GECOS is not inherently epistemically problematic; its normative profile depends on context and use.

A final example concerns *learning and self-explanation*. Users often ask LLMs to explain a concept “like I’m five” or to provide an intuitive overview. In many cases, the resulting explanation is simplified to the point of distortion, yet users still report that it helped them orient themselves. What the shortcut provides here is not mastery but a starting point, a provisional scaffold that lowers the threshold for engagement. The danger arises when the scaffold is mistaken for the structure itself. Again, GECOS clarifies the mechanism: the model supplies a connectable narrative that stabilizes initial sense-making, but deeper understanding requires additional resources and critical engagement that lie outside the shortcut.

Across these examples, several common patterns emerge. First, LLM outputs are rarely treated as final products; they are treated as intermediate artifacts that support further action. Second, interaction tends to be iterative, with users refining prompts rather than evaluating outputs in isolation. Third, responsibility consistently shifts toward the user as soon as selection, integration, or action is involved. These patterns are difficult to explain with metaphors that frame LLMs primarily as epistemic failures or deceptive speakers. They are much more naturally captured by the idea of cognitive shortcuts that reduce friction in moving from uncertainty to a workable next step.

At the same time, the examples also reveal the limits of GECOS. Shortcuts do not discriminate between contexts in which procedural plausibility is sufficient and those in which robust grounding is required. Without external constraints—such as verification tools, institutional norms, or user training—the same mechanism that enables fluent continuation can also stabilize poorly founded decisions. This is not a flaw of the framework but one of its key insights: by identifying the shortcut function, GECOS makes it possible to ask where shortcuts should be supported, where they should be constrained, and where they should be actively discouraged.

## 7 Discussion

GECOS reframes interaction with LLMs by shifting attention away from questions of internal understanding or epistemic deficiency and toward the functional role LLMs play in contemporary communicative practices. Rather than asking whether LLMs “really mean” or “really know,” GECOS asks how their outputs stabilize continuation, reduce friction, and reorganize responsibility under conditions of uncertainty. This shift has several implications—conceptual, normative, and ethical—that merit explicit discussion.

First, GECOS clarifies why debates that focus exclusively on truth, hallucination, or deception risk missing what is most distinctive about LLM use in practice. As shown throughout the paper, many interactions with LLMs are not primarily epistemic in the strong sense. They are compositional, exploratory, or coordinative. In these contexts, success is not measured by correspondence to an external state of affairs but by whether an output enables further action: writing, deciding, reframing, or continuing a conversation. By conceptualizing LLMs as operators for cognitive shortcuts, GECOS makes it possible to acknowledge this pragmatic success without conflating it with understanding or authority. At the same time, it helps explain why purely deficit-based metaphors such as bullshit feel both compelling and insufficient: they correctly diagnose epistemic risk but fail to account for functional integration.

Second, the proposed lens foregrounds the redistribution of responsibility that accompanies shortcut-based interaction. Because LLMs provide connectable continuations rather than commitments, responsibility for meaning, correctness, and consequence does not disappear, it migrates. It shifts toward users and, increasingly, toward the socio-technical infrastructures that constrain and evaluate outputs. This redistribution is often obscured by the conversational interface, which encourages anthropomorphic interpretations. GECOS counters this tendency by insisting on an operational reading: the model does not “say” anything in the strong sense; it offers selections that users may take up. Responsibility emerges at the point of selection, integration, and enactment, not at the point of generation.

This redistribution also sheds light on the risk of normative flattening discussed earlier. When connectability becomes the dominant criterion for success, there is a tendency to privilege fluency, familiarity, and stylistic adequacy over epistemic depth or argumentative rigor (Costello, 2024). GECOS does not treat this as a moral failure of users or designers, but as a structural consequence of shortcut-based interaction. Cognitive shortcuts are valuable precisely because they lower thresholds; the cost is that they may also lower standards unless counterbalanced by institutional norms, verification practices, or explicit user training. From this perspective, the ethical question is not whether shortcuts should exist, but where and how their use should be constrained.

A particularly significant implication concerns authorship. Traditional notions of authorship presuppose that an author is capable, at least in principle, of adequately formulating their thoughts in language. Even when assistance is used (editors, peers, tools), the author is still understood as the primary originator of form and content. LLM-mediated writing unsettles this assumption. Under GECOS conditions, authorship increasingly consists not in producing every formulation oneself, but in providing initial input, iterative interventions, and final selection over a space of machine-generated continuations. What matters is less the ability to phrase everything independently than the ability to guide, constrain, and authorize the shortcut.

Another limitation is the under-explored emotional and aesthetic dimension of LLM interactions. While GECOS accounts for functional coherence, it does not capture how users engage affectively with the LLM; how it becomes a companion, tutor, or object of projection. The experience of fluency and responsiveness can produce emotional bonding, even when users intellectually recognize the absence of sentience. This underscores the need to complement cognitive metaphors like GECOS with phenomenological and psychological accounts that address the felt reality of interaction (Maeda and Quan-Haase, 2024). For example, in contexts of loneliness, learning, or self-reflection, users may derive emotional support from interactions that are technically shallow but experientially rich (Liang et al., 2024).

This shift raises a genuine ethical–philosophical question: at what point does authorship dissolve into mere attribution? If an individual provides only a minimal initial prompt and accepts an LLM’s output with little modification, can they still reasonably claim authorship? The analogy of a teacher claiming authorship of all a student’s work on the basis of a single instruction captures the tension well. The teacher’s sentence may have been necessary, but it is not sufficient to ground authorship of the result. Similarly, if LLM use moves toward a regime in which the initial input alone is treated as sufficient to claim authorship, the concept risks becoming vacuous.

Such a reconceptualization also has institutional implications. Academic integrity, creative ownership, and professional responsibility are still largely governed by models that assume a tight coupling between author and text. GECOS suggests that this coupling is loosening. Future norms may need to distinguish between different modes of authorship: curatorial authorship, directive authorship, and generative authorship, each with different thresholds of responsibility and credit. The framework does not resolve these normative questions, but it provides a vocabulary for articulating them without reverting to moral panic or technological determinism.

In conclusion, the GECOS framework positions LLMs as neither deceptive pseudo-agents nor empty simulators, but as operators that restructure how sense is produced, stabilized, and owned. It highlights both the productivity of cognitive shortcuts and the ethical tensions they generate, especially around authorship, responsibility, and evaluation. Rather than offering closure, the framework invites ongoing reflection on how much delegation we are willing to accept, and under what conditions we still recognize ourselves as authors of the meanings we put into the world.

## Data Availability

The original contributions presented in the study are included in the article/supplementary material, further inquiries can be directed to the corresponding author.
